# Advancing Environmental Justice through Chemical Education: Perspective from New Orleans’ Water and Air Quality Challenges

**DOI:** 10.1021/acs.jchemed.5c01450

**Published:** 2026-03-23

**Authors:** Ja’Lynn Keller, Sita Aggarwal, Rami A. Al-Horani, Morewell Gasseller, Navneet Goyal

**Affiliations:** † Department of Chemistry, 5785 Xavier University of Louisiana, New Orleans, Louisiana 70125,United States; ‡ Department of Chemistry, 3722 Southeastern Louisiana University, Hammond, Louisiana 70402, United States; § Divison of Pharmaceutical Sciences, College of Pharmacy, Xavier University of Louisiana, New Orleans, Louisiana 70125, United States; ∥ Department of Physics, Xavier University of Louisiana, New Orleans, Louisiana 70125, United States

**Keywords:** Undergraduate Research, Water Analysis, Air Analysis, Collaboration, Environmental Justice, Cancer Alley

## Abstract

New Orleans, Louisiana, faces profound environmental vulnerabilities arising from the convergence of industrial pollution, urban runoff, and climate change–driven weather events. This paper examines systemic challenges affecting water and air quality in the region, with particular focus on low-income and marginalized communities disproportionately burdened by environmental degradation. Drawing on educational and community-based monitoring initiatives at Xavier University of Louisiana and Southeastern Louisiana University, we highlight the integration of student learning with real-time data collection of contaminants such as fecal coliforms, heavy metals, hydrocarbons, and polycyclic aromatic hydrocarbons (PAHs). The aftermath of Hurricane Katrina revealed significant fecal contamination in sediment layers, underscoring the enduring environmental consequences of extreme storm events. The crisis of environmental injustice is further exemplified by the industrial corridor known as *Cancer Alley*, where cancer risk from air pollution remains among the highest in the nation. We argue that chemical education can play a critical role in advancing environmental justice through student-centered monitoring, public engagement, and advocacy for cleaner and more equitable infrastructure.

## Graphical abstract

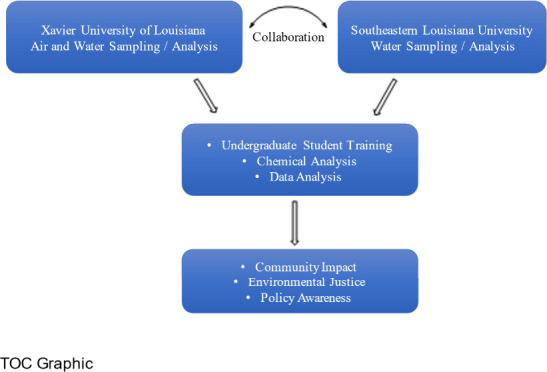

## 1 Introduction

Water is indispensable to life, yet access to clean and safe water remains elusive for many communities. This inequity—commonly described as **water injustice**—reflects broader patterns of environmental disparity disproportionately impacting underrepresented and low-income populations. New Orleans, while celebrated for its culture and resilience, also represents a cautionary tale of environmental vulnerability.^1,2^ Louisiana’s waterways are increasingly compromised by industrial runoff, urbanization, and the lingering effects of climate-related disasters. As a geographically low-lying, coastal city, New Orleans is particularly susceptible to hurricanes, flooding, and infrastructure failure—factors that often lead to widespread water and air contamination.^3–5^ Evidently, Hurricane Katrina of 2005, which devastated southeast Louisiana, was one of the worst environmental disasters in modern American history.^6,7^

This paper examines the contributing factors to environmental degradation in the New Orleans region, highlights ongoing collaborative educational and scientific initiatives to monitor and mitigate these challenges, and advocates for a justice-centered approach to environmental policy and chemical education. We briefly describe two activities that have been initiated over the past two years. The first focuses on air quality monitoring throughout the greater New Orleans region, and the second involves checking water contamination in the New Orleans area in collaboration with Southeastern University, Hammond, Louisiana.

## 2 Water Quality Challenges in New Orleans

The urban infrastructure of New Orleans accelerates the transport of pollutants—ranging from hydrocarbons to pesticides—into its waterways during heavy rainfall.^8,9^ Petrochemical industries along the Mississippi River further exacerbate the problem through the discharge of untreated or partially treated effluents.^3–5,10^ The aftermath of Hurricane Katrina in 2005 revealed how fragile and underprepared the region’s environmental infrastructure truly is. Overwhelmed sewer systems allowed fecal matter, heavy metals, and volatile organic compounds to contaminate ecosystems.^11–13^

Notably, research in Violet Marsh post-Katrina revealed coprostanol levels comparable to highly polluted international sites.^3,14^ Coprostanol is a steroid derived from cholesterol, formed by gut bacteria in mammals. It is the primary odorous compound in feces, making it a key biomarker for sewage contamination in water and soil. Louisiana’s oil refineries continue to be among the top national contributors to waterborne pollution. Released hydrocarbons inhibit aquatic photosynthesis, destabilize fragile ecosystems, and introduce persistent carcinogens such as polycyclic aromatic hydrocarbons (PAHs).^4,15^

Interestingly, although the New Orleans Carrollton Waterworks, which serves 291,044 people using surface water, was in compliance with federal drinking water standards between April and June 2024, testing from years 2014–2023 shows that 12 of the 34 detected contaminants exceeded the Environmental Working Group’s (EWG; a nongovernmental team of scientists, policy experts, lawyers and communications and data experts who work to reform our nation’s broken chemical safety and agricultural laws) health guidelines, which are more stringent than federal limits that have not been updated in nearly two decades. Major contaminants are found at levels far above EWG recommendations, many of which are linked to cancer or other health risks. While legal compliance suggests the water meets outdated regulatory standards, EWG warns that “legal” does not necessarily mean “safe.^14^

## 3 Air Quality and Health in the Region

In New Orleans, people of color represent the majority of the population. Research suggests that people of color are more likely to be living with one or more chronic conditions that make them more susceptible to the health impacts of air pollution.^16–18^ Therefore, air pollution in Southern Louisiana is a significant dimension of the environmental crisis. Emissions from refineries and power plants include sulfur dioxide, nitrogen oxides, and particulate matter—substances linked to increased risks of asthma, cancer, and cardiovascular disease.^16^ These pollutants also contribute to **cross-media contamination**, where air pollutants are deposited into water and soil.^19^

The area known as **Cancer Alley**—a corridor between Baton Rouge and New Orleans—is home to a dense cluster of petrochemical plants.^10,20–22^ The EPA and other watchdog organizations have documented high cancer risk estimates in this area, with disproportionate effects on Black and low-income residents.^23^ Despite mounting evidence, regulatory enforcement has been limited. Legal challenges, such as those targeting emissions from the Denka facility (a chemical plant linked to air pollution and cancer risks in the majority-Black region in the center of Louisiana’s Cancer Alley), have failed to drive systemic change.^24^

## 4 Climate-Driven Risk and Infrastructure Weaknesses

The subtropical climate of New Orleans brings frequent heavy rainfall, which routinely overwhelms rivers, lakes, and wetlands.^25,26^ Pollutants emitted into the atmosphere—such as PAHs and volatile organic compounds (VOCs)—often return via precipitation, contaminating aquatic ecosystems and drinking water sources.^27^ Heavy storm events can also mobilize legacy contaminants, including heavy metals and petrochemicals, from surrounding industrial corridors, further compounding water quality challenges.

Such climate-exacerbated infrastructure vulnerabilities demand urgent attention. As climate change increases the frequency and severity of storms, New Orleans faces an escalating environmental health crisis. Without robust interventions in wastewater management, stormwater infrastructure, and pollution monitoring, vulnerable communities will continue to bear disproportionate health burdens from contaminated air and water.^28–30^

## 5 Environmental Justice and Community Engagement

Communities located near industrial zones face elevated risks of cancer, reproductive issues, and respiratory disease.^10,31–37^ These patterns reveal systemic environmental racism and economic marginalization. Yet, local organizations like RISE St. James have mobilized to oppose industrial expansion and advocate for inclusive environmental governance.^23^ Their work exemplifies how civic engagement and grassroots organizing can shift public discourse and influence policy.

Other groups, including the Louisiana Bucket Brigade and Concerned Citizens of St. John, have also played pivotal roles in documenting pollution through citizen science initiatives, collecting air samples, and raising awareness about toxic exposures. Their collaborations with universities and advocacy networks highlight the importance of community-engaged research in holding industries accountable. Beyond opposing harmful developments, these organizations champion sustainable economic alternatives, such as green jobs and investments in renewable energy, which could reduce reliance on petrochemical industries. Such partnerships between residents, advocacy groups, and academic institutions illustrate the transformative power of community engagement in advancing environmental justice. By integrating lived experiences with scientific data, these efforts help bridge knowledge gaps and empower vulnerable communities to participate directly in decision-making processes.^38–42^ Table 1 presents a list of local organizations and their role in addressing environmental issues.

**Table 1:** New Orleans Environmental Organizations—Air, Water, and Pollution Focus

Organization	Category/Focus	Role/What They Do	Geographic Focus	Online Address
Louisiana Bucket Brigade	Air Quality/Pollution	Community air monitoring, citizen science, and advocacy against industrial emissions	Fenceline communities, Cancer Alley, New Orleans	https://labucketbrigade.org
Deep South Center for Environmental Justice (DSCEJ)	Air & Water Quality/Environmental Justice	Research, community engagement, education on pollution impacts, supports families harmed by industrial pollution	New Orleans & Gulf South	https://www.dscej.org
Rise St. James	Air & Water Quality/Advocacy	Grassroots campaigns to fight petrochemical expansion, monitor air and water pollution	St. James Parish, Cancer Alley	https://risestjames.org
Pontchartrain Conservancy	Water Quality/Watershed	Protects Lake Pontchartrain Basin, monitors water quality, and conducts watershed restoration	Greater New Orleans (Lake Pontchartrain Basin)	https://scienceforourcoast.org
Groundwork New Orleans	Urban Restoration/Water & Pollution	Green infrastructure, stormwater management, and neighborhood environmental restoration indirectly improve water quality	New Orleans neighborhoods	https://groundwork-neworleans.org
SOUL Nola (Sustaining Our Urban Landscape)	Urban Forestry/Air & Water Quality	Tree planting to reduce heat, improve air quality, stormwater absorption, and flood mitigation	New Orleans citywide, includes Carrollton/Uptown	https://soulnola.org
The Green Project	Waste Reduction/Pollution Mitigation	Promotes reuse, recycling, and hazardous material collection to reduce landfill and water pollution	New Orleans	https://thegreenproject.org
Glass Half Full	Recycling/Water & Pollution	Converts glass into sand for coastal restoration, reduces landfill pollution	New Orleans	https://glasshalffullnola.org
Lower Nine Center for Sustainable Engagement and Development	Community/Air & Water Quality	Community education and projects addressing environmental health, air and water pollution, and climate resilience	Lower ninth Ward	https://sustainthenine.org
Water Wise Gulf South	Flood Mitigation/Stormwater & Pollution	Green infrastructure and rain gardens reduce flooding, stormwater runoff, and associated water pollution	New Orleans neighborhoods	https://waterwisegulfsouth.org
Alliance for Affordable Energy	Energy/Pollution & Emissions	Advocates for cleaner energy, reduces air pollution from utilities	Louisiana, New Orleans	https://www.all4energy.org
Green Light New Orleans	Energy Efficiency/Air Quality	Installs energy-efficient lighting and promotes sustainability to reduce emissions	New Orleans	https://greenlightneworleans.org
Urban Conservancy	Urban Environment/Air & Water	Promotes green infrastructure, sustainable development, water management, and pollution mitigation	New Orleans	https://www.urbanconservancy.org

## 6 Activities Performed for Continuous Environmental Monitoring and Student Engagement

Here, we present two ongoing air- and water-monitoring activities conducted at Xavier University of Louisiana. Students from both the Department of Chemistry and the Department of Physics are actively involved in these efforts. Xavier University is a historically Black institution, and most students participating in this research come from underrepresented communities. The monitoring activities are conducted in low-income neighborhoods, such as the Gert Town neighborhood and the Lower Ninth Ward district of New Orleans. Gert Town and the Lower Ninth Ward are historically marginalized, predominantly Black New Orleans neighborhoods that suffered catastrophic damage from 2005’s Hurricane Katrina. Both areas continue to face slow recovery, high poverty rates, and significant population loss, with the Lower Ninth Ward’s population remaining at roughly one-third of its pre-2005 level. In addition, a parallel research effort at Southeastern Louisiana University investigates the effects of heavy metals on cellular processes, particularly protein expression. The students participated in joint sampling campaigns that spanned both urban sites in New Orleans and rural or semirural areas in Southeastern Louisiana. The long-term goal of this work is to integrate components of these community-engaged monitoring activities into laboratory-based coursework and enable the students to compare the results of these activities with the Louisiana standards of air quality and water pollution.^43^

The role of mentors was paramount in these activities. In the two activities, mentors guided the selection of student participants and provided training on assembling and using experimental tools for measuring air and water quality. Mentors also demonstrated proper sampling techniques and instructed students on equipment calibration and data recording. Mentors also assisted with data collection throughout the project. At the outset, mentors outlined the study objectives, discussed the significance of the research, and helped students develop research questions and understand the experimental design. During the activity, mentors encouraged critical thinking and teamwork. They also supported data analysis and visualization. Mentors also guided students in preparing presentations. At the end of each activity, mentors and students discussed the results and their environmental implications.

### Activity 1: Engaging Students and the Community in Air Quality Monitoring Using Low-Cost PM Sensors

This activity engages undergraduate students in monitoring air quality across underserved neighborhoods in New Orleans using low-cost, custom-designed sensors to measure PM_2.5_ and other environmental parameters. Each semester, 3–4 undergraduate research students are recruited and supported through stipends. Students receive hands-on training in sensor assembly, installation, maintenance, data management, and community communication. All students were African American students from underserved communities. The two genders were equally represented. All students had average or above-average academic performance.

Sensors are typically mounted behind the homes (outside) of volunteer families in the New Orleans area to capture highly localized air-quality patterns that are often missed by regulatory monitoring networks. Students work with multiple sensing platforms, including the homemade ECoSTEM sensor, the commercial PurpleAir (PA) sensor, and the Davis AirLink sensor. All three devices utilize the PMS5003 Plantower particulate matter sensor, allowing students to compare performance across platforms and evaluate how engineering design and environmental conditions influence data quality.^44^

The ECoSTEM project is guided by three primary objectives:

1. To develop microcontroller-based systems for collecting environmental data—primarily airborne particulate matter—and deploy them across the New Orleans region.
2. To engage Xavier University undergraduates in partnerships with public school teachers, high school students, and government agencies to apply STEM approaches to real-world environmental challenges.
3. To expand STEM educational practices for faculty and students through hands-on, community-centered research experiences.

Two versions of the ECoSTEM sensor are used: (i) a benchtop version equipped with an OLED display for classroom instruction and high school workshops, and (ii) a field-deployment version in which the OLED is replaced by a data logger and SD card to enable long-term outdoor monitoring. This dual design provides students with experience in sensor fabrication, electronics, calibration, and extended environmental data collection.

Each deployed sensor contains a chip-based data card that is collected weekly over a 3–6 month period, generating high-resolution spatial and temporal data sets. The project workflow includes field deployment, routine instrument checks, data retrieval, and preliminary quality control. To promote reproducibility, sensor assembly instructions, deployment protocols, and representative data sheets can be provided as Supporting Information to facilitate adaptation by other educators.

Below (Figure 1) are depictions of our sensor. In the figure, “1a” shows the assembly of the sensor, “1b” shows the installation behind the wall, “1c” shows another outdoor assembly of the sensor and continuous solar power, and “1d” shows a big sensor on top of the building of XULA.

**Figure 1:**
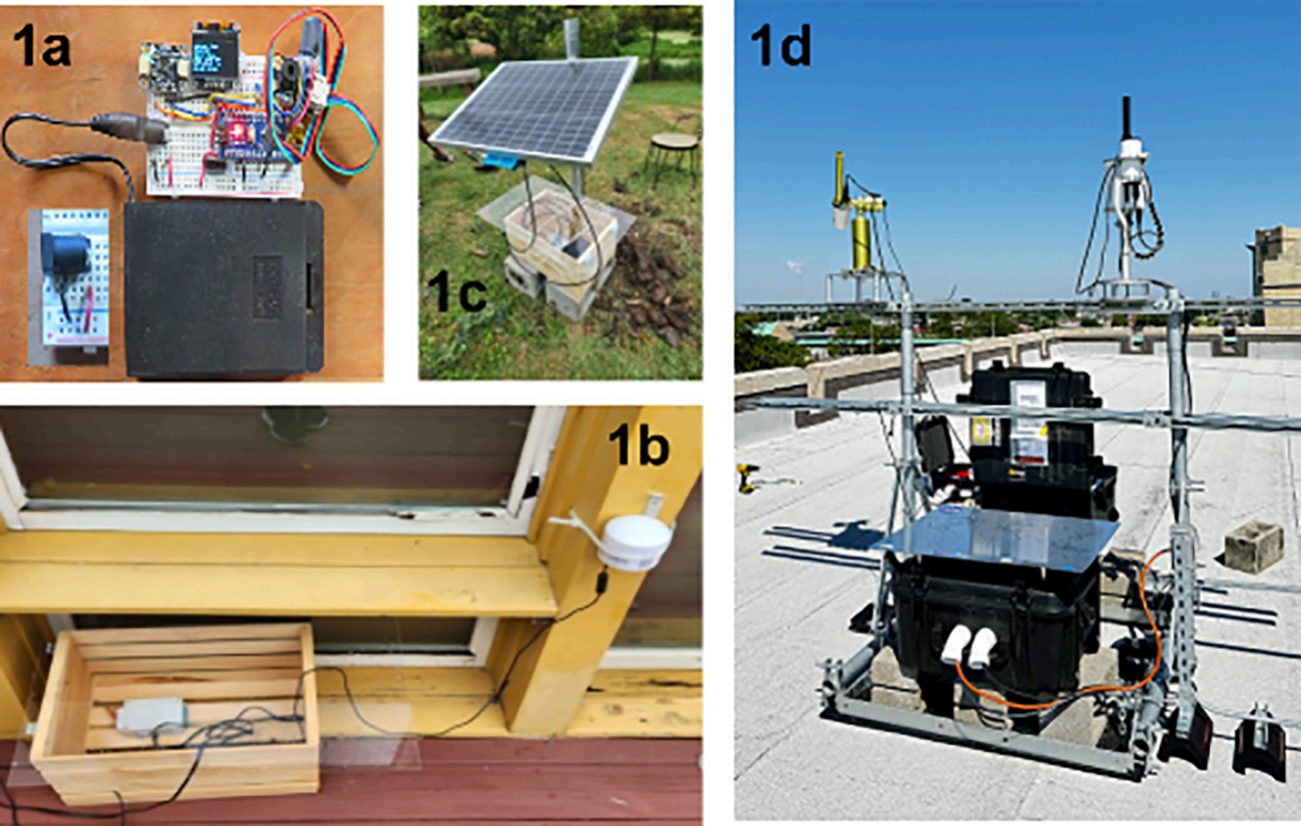
From left to right: (1) Schematic of an Arduino-based environmental monitoring device equipped with ambient temperature, relative humidity, and surface-temperature sensors; (2) a homemade PM_2_._5_ sensor collocated with a PurpleAir unit deployed in the Lower Ninth Ward of New Orleans; (3) a solar-powered homemade PM_2_._5_ sensor installed at a site lacking access to grid electricity; and (4) a rooftop deployment on the Xavier University campus showing a PurpleAir and a Davis AirLink sensor collocated with NASA’s AERONET and PANDORA instruments, enabling long-term, multiplatform air-quality measurements.

While low-cost particulate matter (PM) sensors significantly expand monitoring capacity, they require careful calibration, correction for humidity and temperature effects, and ongoing maintenance. These challenges are intentionally integrated into student training to strengthen technical and analytical skills. A continued area of development focuses on designing sensor systems capable of continuous, long-term monitoring with minimal data loss and greater operational stability. In addition to characterizing particle size distributions, we are currently developing laboratory-based experiments to chemically and physically analyze particles captured by these sensors.

### Activity 2: Water Monitoring in Collaboration with Parish Water Board and Other Regional Universities

In a complementary activity focused on water contamination, undergraduate students from the Department of Chemistry participate in community-based water sampling and analysis across the Greater New Orleans region. Each semester, students are trained in systematic field collection methods and sample handling, and they collect water from a range of sites, including residential neighborhoods, storm-impacted areas, and locations with aging or vulnerable infrastructure. This fieldwork introduces students to standardized sampling protocols, environmental stewardship, and the complexities of monitoring water quality in urban systems.

The water quality monitoring activity is guided by the following core objectives:

1. To develop and implement systematic, community-based water sampling protocols for the detection of inorganic and organic contaminants.
2. To engage undergraduate students in hands-on environmental research through field sampling, laboratory analysis, and data interpretation.
3. To foster interdisciplinary collaboration by integrating environmental chemistry, toxicology, approaches through partnerships with regional institutions, and water authorities.

At Xavier University of Louisiana, the program is conducted in collaboration with the Jefferson Parish Water Board, which assists with site selection and provides access to relevant municipal data. Students analyze samples for nitrates, total coliform bacteria, and polycyclic aromatic hydrocarbons (PAHs; Figure 2), with a particular focus on changes in contamination levels following major storm events. Recently acquired instrumentation—including Inductively Coupled Plasma (ICP) systems for trace metal analysis and Gas Chromatography–Mass Spectrometry (GC–MS) for organic pollutant detection—enables students to conduct research-grade measurements. Building on these capabilities, the program has initiated preliminary screening for selected per- and polyfluoroalkyl substances (PFAS; Figure 2) in local water sources.^45^

**Figure 2:**
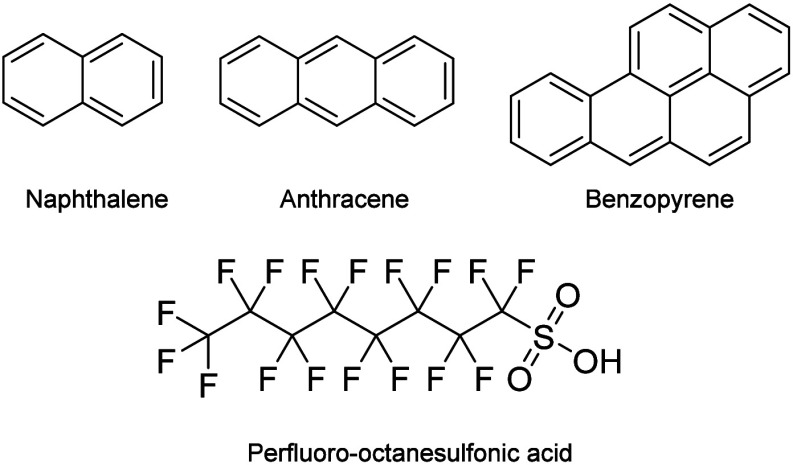
Representative chemical structures of PAHs (polycyclic aromatic hydrocarbons) and PFAS (per- and polyfluoroalkyl substances), which are two distinct classes of persistent, harmful environmental pollutants commonly found in water, soil, and consumer products.

As mentioned earlier, a parallel research effort at Southeastern Louisiana University (SLU) investigates the effects of heavy metals on cellular processes, particularly protein expression.^46^ Through a developing partnership between Xavier and SLU, students participate in joint sampling campaigns that span both urban sites in New Orleans and rural or semirural areas in Southeastern Louisiana. This regional comparison allows students to evaluate spatial differences in contaminant profiles and environmental risk.

The collaboration offers students the opportunity to rotate between campuses, develop advanced analytical skills, and engage with faculty and peers across institutions. By linking environmental chemistry with molecular and toxicological perspectives, the program provides an interdisciplinary framework for understanding pollution and its implications for human health.

Looking forward, the program plans to expand through partnerships with nonprofit organizations working in environmental advocacy, public health, and community resilience. A central goal is to extend educational outreach to high school classrooms by teaching students to interpret basic water-quality indicators and recognize common contaminants in local water sources. By promoting data literacy and environmental awareness among younger learners, the program aims to cultivate a pipeline of informed, empowered future scientists and community leaders.^46^

### Safety Statement

No unexpected or unusually high safety hazards were encountered

## 7 Conclusion

New Orleans serves as a powerful case study in environmental justice, exemplifying how climate change, industrial pollution, and underfunded infrastructure converge to threaten vulnerable communities. Scientific tools like environmental monitoring, when paired with community engagement and inclusive education, offer a way forward. Recent national legal actions—such as the court-approved $10 billion settlement over PFAS contamination—underscore both the scale of chemical pollution and the urgent need for accountability and reform in environmental regulation.^47,48^ These landmark decisions highlight how science, policy, and public advocacy must intersect to address systemic environmental harms.

Chemical education has a critical role to play. By integrating applied environmental research into undergraduate curricula and engaging students in community-relevant projects, educators can cultivate a new generation of scientists committed to sustainability and equity. Hands-on monitoring projects at institutions like Xavier University of Louisiana and Southeastern Louisiana University not only train students in advanced analytical techniques but also expose them to the lived realities of environmental injustice in the Gulf South. Field-based sampling, intercampus collaboration, and partnerships with grassroots organizations transform coursework into civic engagement, helping students understand the direct social consequences of pollution data. This approach echoes national calls for “convergence education,” where science, policy, and justice intersect to prepare students for leadership in addressing global challenges.^49,50^
